# Polygamy and Risk of Coronary Artery Disease in Men Undergoing Angiography: An Observational Study

**DOI:** 10.1155/2017/1925176

**Published:** 2017-01-30

**Authors:** Amin Daoulah, Amir Lotfi, Mushabab Al-Murayeh, Salem Al-kaabi, Salem M. Al-Faifi, Osama E. Elkhateeb, Mohamed N. Alama, Ahmad S. Hersi, Ciaran M. Dixon, Waleed Ahmed, Mohamed Al-Shehri, Ali Youssef, Ahmed Moustafa Elimam, Ayman S. Abougalambou, Waheed Murad, Alawi A. Alsheikh-Ali

**Affiliations:** ^1^Section of Adult Cardiology, Cardiovascular Department, King Faisal Specialist Hospital & Research Center, Jeddah, Saudi Arabia; ^2^Division of Cardiology, Baystate Medical Center, Tufts University School of Medicine, Springfield, MA, USA; ^3^Cardiovascular Department, Armed Forces Hospital, Southern Region, Khamis Mushayt, Saudi Arabia; ^4^Cardiovascular Department, Zayed Military Hospital, Abu Dhabi, UAE; ^5^Section of Pulmonology, Internal Medicine Department, King Faisal Specialist Hospital & Research Center, Jeddah, Saudi Arabia; ^6^Cardiac Center, King Abdullah Medical City in Holy Capital, Makkah, Saudi Arabia; ^7^Cardiovascular Department, King Abdulaziz University Hospital, Jeddah, Saudi Arabia; ^8^Cardiovascular Department, College of Medicine, King Saud University, Riyadh, Saudi Arabia; ^9^Emergency Department, King Faisal Specialist Hospital & Research Center, Riyadh, Saudi Arabia; ^10^Section of Infectious Disease, Internal Medicine Department, King Faisal Specialist Hospital & Research Center, Jeddah, Saudi Arabia; ^11^Cardiovascular Department, Suez Canal University, Ismailia, Egypt; ^12^College of Medicine, Mohammed Bin Rashid University of Medicine and Health Sciences, Dubai, UAE; ^13^Institute of Cardiac Sciences, Sheikh Khalifa Medical City, Abu Dhabi, UAE

## Abstract

Epidemiologic evidence suggests a link between psychosocial risk factors such as marital status and coronary artery disease (CAD). Polygamy (multiple concurrent wives) is a distinct marital status practiced in many countries in Asia and the Middle East, but its association with CAD is not well defined. We conducted a multicenter, observational study of consecutive patients undergoing coronary angiography during the period from April 1, 2013, to March 30, 2014. Of 1,068 enrolled patients, 687 were married men. Polygamy was reported in 32% of married men (1 wife: 68%, 2 wives: 19%, 3 wives: 10%, and 4 wives: 3%). When stratified by number of wives, significant baseline differences were observed in age, type of community (rural versus urban), prior coronary artery bypass grafting (CABG), and household income. After adjusting for baseline differences, there was a significant association between polygamy and CAD (adjusted OR 4.6 [95% CI 2.5, 8.3]), multivessel disease (MVD) (adjusted OR 2.6 [95% CI 1.8, 3.7]), and left main disease (LMD) (adjusted OR 3.5 [95% CI 2.1, 5.9]). Findings were consistent when the number of wives was analyzed as a continuous variable. In conclusion, among married men undergoing coronary angiography for clinical indications, polygamy is associated with the presence of significant CAD, MVD, and LMD.

## 1. Introduction

Coronary artery disease (CAD) is a leading cause of morbidity and mortality worldwide. In addition to the conventional risk factors for CAD, there is increasing awareness of the role played by psychosocial determinants such as marital status. It is well described that marriage offers health benefits and is associated with lower risk of all-cause mortality and lower risk of ischemic heart disease in men; however, the underlying mechanism is not well understood and several hypotheses have been proposed [1–8]. The majority of studies examining the relationship between marital status and CAD were conducted in western societies or developed countries, and none examined the role of multiple concurrent marriages (polygamy). Polygamy is a distinct marital status practiced in many developing countries, including those in Asia and the Middle East, but its association with CAD is not well defined. We therefore conducted an observational study examining the association between polygamy and the presence of significant CAD among men undergoing coronary angiography for clinical indications.

## 2. Methods

### 2.1. Patient Population

This is a multicenter, multiethnic, cross-sectional observational study. Data were collected prospectively from five hospitals in two Gulf regions (the Kingdom of Saudi Arabia and the United Arab Emirates), during the period from April 1, 2013, to March 30, 2014. The study was approved by King Faisal Specialist Hospital & Research Center Institutional Review Board, and an invitation letter was given to all participants who affirmed verbal consent prior to their enrollment. For each patient undergoing coronary angiography for a clinical indication, two separate data forms, one general and one angiographic, were filled out by the research assistant and assigned cardiologist, respectively (Supplementary Material available online at https://doi.org/10.1155/2017/1925176). Both forms were completed before the patients were discharged from the hospital. All data forms were reviewed by the assigned cardiologist and then sent online to the principle investigator, who also checked the forms before submission for analysis.

The general data form recorded the following information:

- Demographic data: age, gender, and ethnic background
- Physiologic status: history of hypertension, diabetes, or dyslipidemia and body mass index
- Life style: smoking history (current/ex-smoker)
- Past medical history: coronary artery disease, percutaneous coronary intervention, coronary artery bypass surgery, cerebral vascular disease, peripheral arterial disease, congestive heart failure, atrial fibrillation, or chronic kidney disease
- Socioeconomic data: occupation (unemployed, private sector, government sector, and self-employed), living in rural or urban area, last level of education completed (illiterate, secondary school, university, masters, or doctorate), monthly income (<1300, 1300 to 2600, 2600 to 5300, 5300 to 7900, 7900 to 10600, and >10600 US dollars)
- Ethnicity: Arabic from Gulf region, Arabic from non-Gulf region, and non-Arabic.
- Current marital status (number of wives): One wife or polygamy (multiple concurrent wives)

The angiographic data form recorded the following:

- Reason for coronary angiography (elective versus urgent/emergent)
- Number of vessels involved (severity)
- Treatment (medical versus revascularization)

All patients who underwent coronary angiography were eligible for the study. There were no exclusion criteria.

### 2.2. Definitions

Significant coronary artery disease was defined as ≥70% luminal stenosis in a major epicardial vessel or ≥50% stenosis in the left main coronary artery. Multivessel disease (MVD) was defined as having more than one significant coronary artery disease.

### 2.3. Statistical Analysis

Standard summary statistics were used to describe the cohort. Continuous variables are presented as mean ± standard deviation and were compared across multiple groups using the analysis of variance test. Categorical variables are presented as percentages and compared using the Chi-square test. The association between polygamy and CAD was examined using logistic regression with and without adjustment for baseline differences. *P* < 0.05 was considered statistically significant.

## 3. Results

### 3.1. Overall Patients Baseline Characteristics

We enrolled 1,068 patients, 687 (64%) were married men who underwent a coronary angiogram for elective (48%) or nonelective (52%) indications. Nonelective indications included non-ST elevation acute coronary syndrome (NSTEACS) (46%) and ST elevation acute myocardial infarction (STEMI) (6%). They were relatively young (age 59 ± 12), predominantly of Arab Gulf origin (87%), and from an urban setting (73%). Comorbid conditions including conventional risk factors for cardiovascular disease were common, most notably dyslipidemia (66%), hypertension (57%), diabetes mellitus (56%), and current or past smoking (54%). Many had a prior history of CAD (45%) or coronary revascularization (percutaneous coronary intervention [PCI] in 24% or coronary artery bypass graft surgery [CABG] in 6%). Other reported manifestations of cardiovascular or associated comorbid conditions included atrial fibrillation (5%), congestive heart failure (13%), cerebrovascular disease (4%), chronic kidney disease (14%), depression (8%), and peripheral vascular disease (2%). Nearly half of the patients were illiterate (42%). Most patients (80%) reported income levels of less than the equivalent of 32,000 USD annually, and 21% were not employed at the time of the study (Table 1).

### 3.2. Coronary Angiogram Findings

Coronary angiogram revealed significant coronary artery disease in 72% of patients, multivessel disease in 48%, and left main disease in 12%. Consequently, 47% underwent PCI and 17% CABG during the index admission, and the remainder (36%) were treated with medical therapy alone (Table 2).

### 3.3. Polygamy and CAD

**Figure 1:**
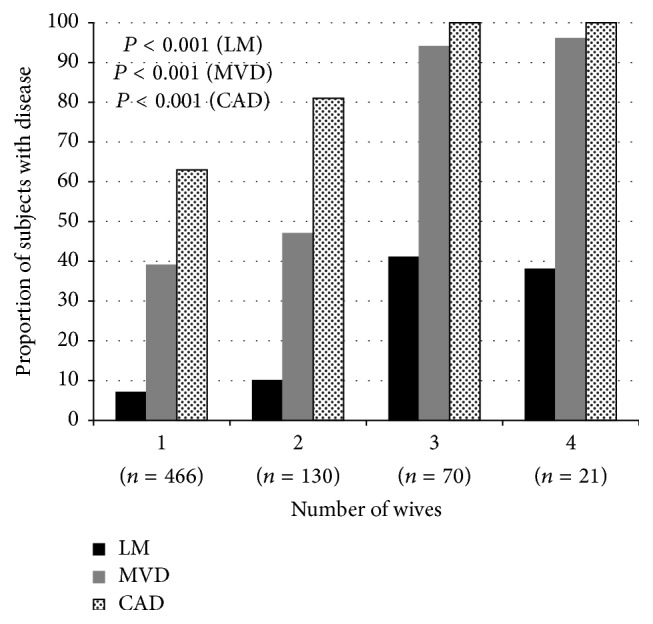
Relationship between number of wives and coronary angiogram findings.

Married men (*n* = 687) were categorized according to number of wives, and nearly one-third were polygamous. The majority had one wife (68%), and the remainder had two wives (19%), three wives (10%), or four wives (3%). Men with more than one wife were more likely to be older, live in a rural area, have a higher income level, and have a history of prior CABG (Table 1). With increasing number of wives, there were a higher proportion of men with CAD, MVD, or LMD (Figure 1). After adjusting for baseline differences, there was a significant association between polygamy and CAD (adjusted OR 4.6 [95% CI 2.5, 8.3]), MVD (adjusted OR 2.6 [95% CI 1.8, 3.7]), and LMD (adjusted OR 3.5 [95% CI 2.1, 5.9]) (Table 3). Findings were consistent when the number of wives was analyzed as a continuous variable (Table 3).

## 4. Discussion

Numerous studies have shown that marriage offers better overall health and mortality outcomes [1–8]. Also, it has been shown that remarried men do not differ in health outcome compared to men in a lifelong marriage [9–11]. On the other hand, the influence of polygamy (a distinct marital status of multiple concurrent wives) on cardiac disease has not been studied. To the best of our knowledge, the present study is the first to assess the association between polygamy and the severity and number of coronary artery lesions. Among men undergoing coronary angiography for clinical indications, a polygamous relationship was associated with 2 to 4 times higher odds of CAD, multivessel disease, or left main disease. This association persisted after adjusting for baseline differences and when the number of wives was treated as a continuous variable. In our cohort population, polygamy was more frequent in rural areas because it is more culturally acceptable, and getting married at a young age is more common in these areas. Men with multiple wives have to be well supported financially, and although Saudis and Emirati citizens are supported by their governments, polygamists may need more than one income. They may therefore take on extra employment or have the added pressure of travelling daily to urban areas for higher paid work.

A number of possible mechanisms may contribute to the association between polygamy and the severity and number of coronary artery lesions. It could be that the need to provide and maintain separate households multiplies the financial burden and emotional expense of polygamy. Each household must be treated fairly and equally, and it is likely that the stress of doing that for several spouses and possibly several families of children is considerable [12, 13]. Also, psychosocial factors may play a role in making them less compliant with their cardiac medication and having less time for physical activity, which could be relevant in those with prior CABG [14–17]. It is also possible that biological mechanisms related to the stress of polygamy can significantly lower testosterone levels secondary to increased cortisol levels [18–20]. Gray et al. looked at the relationship between testosterone levels and marriage among Ariaal men of Northern Kenya who are considered aloof spouses and provide minimal parenting. They found that men with more than one wife (polygynously married men) had lower levels of testosterone compared to their monogamously married counterpart [21]. Also, stress related to polygamy may have a role in blood pressure reactivity, increased hemoglobin A1C, reduced sleep time, impairment of efforts to be physically active, and poor dietary habits [6, 22–27]. In addition, the sympathetic nervous system may be activated, resulting in hemodynamic changes that may cause increased vascular resistance, transient myocardial ischemia, and/or disruption of vulnerable coronary plaques. In addition, stress may have proinflammatory and prothrombotic effects [28]. This study provides us with new information regarding the association between CAD and polygamy in a Gulf population. A larger study is required to confirm these findings. Future studies should involve additional regions and a more racially diverse group of subjects. Study designs aimed at elucidating potential mechanisms underlying these associations as well as the effect of polygamy on women's coronary risk should be sought.

The strengths of this study are that it is the first to look at the association between distinct marital status (polygamy) and CAD using coronary angiography in a Gulf population.

We acknowledge a number of limitations. First, our study had a small sample size. Second, the time interval from being married with more the one wife to the cardiac catheterization was not recorded; this interval may have an influence on the findings. Third, our study population was selected to undergo coronary angiography if clinically indicated and as such cannot be generalized to all men who practice polygamy in the Gulf region. In the absence of reliable statistics on the prevalence of polygamy in the region, it is not clear if the prevalence we observed in our sample is significantly different from that of the general population. Fourth, adjustment for measured variables (e.g., age) may not have completely accounted for their potential confounding effects, and unmeasured confounding variables such as dietary habits, medication adherence, number of children, physical activity, or other unconsidered variables may also have influenced the association.

## 5. Conclusion

Among married men undergoing coronary angiography for clinical indications, polygamy is associated with the presence of significant CAD, MVD, and LMD.

## Supplementary Material

Supplementary material includes a general data form, and an angiographic data form.

## Figures and Tables

**Table 1:** Overall patients baseline characteristics stratified by their number of wives.

	All (*n* = 687)	1 wife (*n* = 466)	2 wives (*n* = 130)	3 wives (*n* = 70)	4 wives (*n* = 21)	*P* value
Age	59 ± 12	58 ± 13	60 ± 11	61 ± 10	59 ± 13	0.01
BMI	28 ± 6	27 ± 6	28 ± 6	28 ± 6	27 ± 8	0.48
Rural (%)	27	24	32	36	43	0.027
DM (%)	56	55	58	61	48	0.726
Hypertension (%)	57	54	65	61	57	0.147
Smoking (%)	54	55	51	57	48	0.613
Dyslipidemia (%)	66	65	64	79	67	0.136
Past history (%)						
CAD	45	42	51	51	43	0.224
PCI	24	23	29	20	14	0.264
CABG	6	4	12	13	10	0.001
AF	5	5	5	4	0	0.746
CHF	13	12	15	13	14	0.848
CVA	4	3	5	9	5	0.134
CKD	14	13	15	23	10	0.13
Depression	8	9	6	7	5	0.666
PAD	2	2	4	4	0	0.199
Ethnicity (%)						0.215
Arabic Gulf region	87	86	90	86	100	
Arabic Non-Gulf	6	7	3	10	0	
Non-Arabic	7	7	7	4	0	
Monthly income (%)						<0.001
<$1300	50	52	49	44	40	
$1300–2600	29	29	26	29	35	
$2600–5300	13	14	12	10	10	
$5300 to 7900	4	3	9	7	0	
$7900 to 10600	2	1	2	6	5	
>$10600	2	1	2	4	10	
Job category (%)						0.127
Jobless	21	22	20	21	19	
Private	18	17	20	20	10	
Government	43	40	46	50	62	
Self-employee	18	21	14	9	9	
Education level (%)						0.778
Illiterate	42	41	43	43	38	
Secondary school	38	39	38	34	33	
Postgraduate	16	15	16	16	29	
Masters	3	4	3	4	0	
Ph.D.	1	1	0	3	0	

DM, diabetes mellitus; CAD, coronary artery disease; BMI, body mass index; CAD, coronary artery disease; PCI, percutaneous coronary intervention; CABG, coronary artery bypass grafting; AF, atrial fibrillation; CHF, congestive heart failure; CVA, cerebrovascular accident; CKD, chronic kidney disease; PAD, peripheral arterial disease; $, USA dollars; Ph.D., a doctor of philosophy.

**Table 2:** Coronary angiogram findings stratified by their number of wives.

	All (*n* = 687)	1 wife (*n* = 466)	2 wives (*n* = 130)	3 wives (*n* = 70)	4 wives (*n* = 21)	*P* value
Indication for CAG (%)						0.062
Elective	48	52	44	30	33	
NSTEACS	46	42	50	63	57	
STEMI	6	6	6	7	10	
Findings on CAG (%)						<0.001
No CAD	28	37	19	0	0	
Single vessel disease	24	24	34	6	4	
Double vessel disease	26	28	30	11	10	
Triple vessel disease	22	11	17	83	86	
Multivessel disease	48	39	47	94	96	<0.001
Left main disease	12	7	10	41	38	<0.001
Intervention (%)						<0.001
Medical therapy	36	39	45	3	5	
PCI	47	53	49	16	14	
CABG	17	8	6	81	81	

CAD, coronary artery disease; STEMI, ST segment elevation myocardial infarction; NSTEACS, non-ST-segment elevation acute coronary syndromes; CAG, coronary angiography; PCI, percutaneous coronary intervention; CABG, coronary artery bypass grafting.

**Table 3:** Association of number of wives and polygamy with coronary artery disease in univariate and multivariate logistic regression.

	Polygamy		Number of wives	
	Univariate	Multivariate^*∗*^	Univariate	Multivariate^*∗*^
Coronary artery disease	4.5 [2.9, 7.2]	4.6 [2.5, 8.3]	3.7 [2.5, 5.4]	4.0 [2.4, 6.6]
Multivessel disease	3.2 [2.3, 4.4]	2.6 [1.8, 3.7]	2.8 [2.2, 3.6]	2.6 [2.0, 3.4]
Left main disease	3.7 [2.3, 5.9]	3.5 [2.1, 5.9]	2.4 [1.9, 3.1]	2.5 [1.9, 3.2]

^*∗*^Adjusted for age, body mass index, history of smoking, diabetes, dyslipidemia, hypertension, coronary artery disease, percutaneous coronary intervention, coronary artery bypass graft surgery, indication for coronary angiography, income level, and community (rural versus urban).
