# Supplementary material for: Polygamy and Risk of Coronary Artery Disease in Men Undergoing Angiography: An Observational Study
Source: Int J Vasc Med. 2017 Jan 30;2017:1925176. doi: 10.1155/2017/1925176 (PMC5303868; doi:10.1155/2017/1925176)
Supplement: Supplementary file 1 — Supplementary material includes a general data form, and an angiographic data form. [file 1925176.f1.doc]

**General Data Form**

1. **Investigator Name:**

**B. Hospital Name:**

**C. Patient Information:**

1. **Patient Name:**
2. **Hospital File Number:**

1. **Date of Birth:**
2. **Date of Initial Evaluation:**
3. **Ethnicity:**

□ Arabian Gulf region □ Arabian Non-Gulf region □ Non Arabic

**D. Patient Baseline Characteristics:**

| ***Baseline Characteristics*** | | | | | | | | | | |
| --- | --- | --- | --- | --- | --- | --- | --- | --- | --- | --- |
| **Weight ( Kg)** |  | | | | | | | | | |
| **Height (m)** |  | | | | | | | | | |
| **BMI ( Kg /M²)** |  | | | | | | | | | |
| **Smoker** | **YES** | | **EX** | | **NO** | | | | | |
|  | |  | |  | | | | | |
| **Diabetes Mellitus** | **IDDM** | | **NIDDM** | | **NO** | | | | | |
|  | |  | |  | | | | | |
| **Dyslipidemia** | **YES** | | | | **NO** | | | | | |
|  | | | |  | | | | | |
| **Hypertension** | **YES** | | | | **NO** | | | | | |
|  | | | |  | | | | | |
| **H/O of Coronary Heart Disease** | **YES** | | | | **NO** | | | | | |
|  | | | |  | | | | | |
| **H/O of Chronic Kidney Disease** | **YES** | | | | **NO** | | | | | |
|  | | | |  | | | | | |
| **H/O of**  **Peripheral Arterial disease** | **YES** | | | | **NO** | | | | | |
|  | | | |  | | | | | |
| **H/O of Cerebrovascular**  **Accident** | **YES** | | | | **NO** | | | | | |
|  | | | |  | | | | | |
| **H/O of PCI** | **YES** | | | | **NO** | | | | | |
|  | | | |  | | | | | |
| **H/O of CABG** | **YES** | | | | **NO** | | | | | |
|  | | | |  | | | | | |
| **H/O of Atrial Fibrillation** | **YES** | | | | **NO** | | | | | |
|  | | | |  | | | | | |
| **H/O of Congestive Heart Failure** | **YES** | | | | **NO** | | | | | |
|  | | | |  | | | | | |
| **H/O of Depression** | **YES** | | | | **NO** | | | | | |
|  | | | |  | | | | | |
| **Married** | **YES** | | | | **NO** | | | | | |
|  | | | |  | | | | | |
| **Number of Wives Currently Living with Him** | **X1** | | **X2** | | **X3** | | | | **X4** | |
|  | |  | |  | | | |  | |
| **Number of Divorces** | X1 | | X2 | | X3 | | | X4 | | |
|  | |  | |  | | |  | | |
| **Widowhood** | **YES** | | | | **NO** | | | | | |
|  | | | |  | | | | | |
| **Wives Nationality** | **Arabic Gulf Region** | | **Arabic Non-Gulf Region** | | **Non- Arabic** | | | | | |
|  | |  | |  | | | | | |
| **Educational Level** | **Illiterate** | **Secondary School** | | **Post Graduate** | **Master** | | | | **PhD** | |
|  |  | |  |  | |  | | | |
| **Living in Urban or Ruler Area** | **Urban Area** | | | | **Ruler Area** | | | | | |
|  | | | |  | | | | | |
| **Monthly Income** | **< $1300** | **$1300-2600** | | **$2600-5300** | **$5300-7900** | **$7900-10600** | | | | > **$10600** |
|  |  | |  |  |  | | | |  |
| **Job Category** | **Jobless** | | **Private Sector** | | **Government**  **Sector** | | | | **Self-employed** | |
|  | |  | |  | | | |  | |

**Angiographic Data Form**

|  |  | | | | |
| --- | --- | --- | --- | --- | --- |
| Indication for Coronary Angiogram  ( Elective vs Urgent/Emergent ) | Elective | | | Urgent/Emergent | |
|  | | |  | |
| No of Vessel with Luminal Stenosis of 50% or more in a Major Epicardial Vessel other than Left Main Artery | 0 | 1 | | 2 | 3 |
|  |  | |  |  |
| Luminal stenosis of 50% or more in the Left main Artery (Left Main Disease) | Yes | | No | | |
|  | |  | | |
| No of Vessel with Luminal Stenosis of 70% or more in a Major Epicardial Vessel other than Left Main Artery | 0 | 1 | 2 | | 3 |
|  |  |  | |  |
| Coronary Intervention  ( PCI vs CABG ) | PCI | | CABG | | |
|  | |  | | |
| Medical Treatment |  | | | | |

**Physician Name Filling the Form:**
